# Downregulation of miR-542-3p promotes osteogenic transition of vascular smooth muscle cells in the aging rat by targeting BMP7

**DOI:** 10.1186/s40246-019-0245-z

**Published:** 2019-12-11

**Authors:** Huan Liu, Hongwei Wang, Sijin Yang, Dehui Qian

**Affiliations:** 1 Department of Cardiology, Second Hospital Affiliated to the Army Medical University, Xinqiao Hospital, Chongqing, 400037 China; 2 The Precision Medicine Institute, The Third Affiliated Hospital, Southern Medical University, Guangzhou, 510150 Guangdong China; 3 Department of Orthopaedics, The Second Affiliated Hospital of Southwest Medical University, Lu Zhou, 646000 Sichuan China; 4 Department of Cardiology and Neurology, The Second Affiliated Hospital of Southwest Medical University, 184 Chunhui Street, Lu Zhou, 646000 Sichuan China; 5 Department of Orthopedics, General Hospital of Shenyang Military Area Command of Chinese PLA, Shenyang, 110016 Liaoning China

**Keywords:** Mir-542-3p, Vascular smooth muscle cells, Osteogenic differentiation, Aging

## Abstract

**Background:**

Aging is believed to have a close association with cardiovascular diseases, resulting in various pathological alterations in blood vessels, including vascular cell phenotypic shifts. In aging vessels, the microRNA(miRNA)-mediated mechanism regulating the vascular smooth muscle cell (VSMC) phenotype remains unclarified. MiRNA microarray was used to compare the expressions of miRNAs in VSMCs from old rats (oVSMCs) and young rats (yVSMCs). Quantitative reverse transcription real-time PCR (qRT-PCR) and small RNA transfection were used to explore the miR-542-3p expression in oVSMCs and yVSMCs *in vitro*. Calcification induction of yVSMCs was conducted by the treatment of β-glycerophosphate (β-GP). Alizarin red staining was used to detect calcium deposition. Western blot and qRT-PCR were used to investigate the expression of the smooth muscle markers, smooth muscle 22α (SM22α) and calponin, and the osteogenic markers, osteopontin (OPN), and runt-related transcription factor 2 (Runx2). Lentivirus was used to overexpress miR-542-3p and bone morphogenetic protein 7 (BMP7) in yVMSCs. Luciferase reporter assay was conducted to identify the target of miR-542-3p.

**Results:**

Compared with yVSMCs, 28 downregulated and 34 upregulated miRNAs were identified in oVSMCs. It was confirmed by qRT-PCR that oVSMC expressed four times lower miR-542-3p than yVSMCs. Overexpressing miR-542-3p in yVSMCs suppressed the osteogenic differentiation induced by β-GP. Moreover, miR-542-3p targets BMP7 and overexpressing BMP7 in miR-542-3p–expressing yVSMCs reverses miR-542-3p’s inhibition of osteogenic differentiation.

**Conclusions:**

miR-542-3p regulates osteogenic differentiation of VSMCs through targeting BMP7, suggesting that the downregulation of miR-542-3p in oVSMCs plays a crucial role in osteogenic transition in the aging rat.

## Background

Aging is believed to have a close association with cardiovascular diseases [24]. In elderly patients, the outcomes of vascular angioplasty and stenting are poorer than those in younger patients [3] and also with a fairly higher risk of complication [10, 29]. Vascular aging causes various pathological alterations in blood vessels, including chronic inflammation, vascular cell phenotypic shifts, and structural modifications, which lead to remodeling of the vascular wall with increased thickness and stiffness and drive arteriosclerosis and atherosclerosis [16].

Vascular smooth muscle cells (VSMCs) are contractile cells found in blood vessels and express smooth muscle markers, including smooth muscle α-actin (SM α-actin), smooth muscle 22α (SM22α), and calponin. VSMCs are not terminal differentiated cells, which can switch from the contractile phenotype to the synthetic phenotype in response to local cues and give rise to the development of aberrant remodeling of vessels [7]. Increasing evidence indicates that aging is a crucial factor promoting the phenotypic shift of VSMCs and results in aging-related deterioration of blood vessels [17, 25]. The dedifferentiation of VSMCs into osteoblast-like cells has been demonstrated to be associated with vascular calcification in the vessel wall [7]. The osteogenic transition of VSMCs is accompanied by the gain of osteogenic markers, such as runt-related transcription factor 2 (Runx2), osteopontin (OPN), and alkaline phosphatase [7]. The triggers of the osteogenic transition of VSMCs have been extensively studied, and many risk factors are recognized, including the loss of calcification inhibitors, senescence, cell death and damage, oxidative stress, mechanical stress, and mitochondrial dysfunction [7]. However, in aging vasculature, the mechanism driving the osteogenic transition of VSMCs remains unclarified.

MicroRNAs (miRNAs) are non-coding, single-stranded RNA molecules and function in RNA silencing and post-transcriptional regulation of gene expression, regulating a number of physiological and pathological processes. Within the cardiovascular system, altered miRNA expression has been found in the blood of patients [2], and certain miRNAs have been found to be involved in various cardiovascular diseases, such as myocardial infarction, arrhythmias, hypertension, and atherosclerosis [33]. In the vascular wall, aging is associated with endothelial dysfunction and a heterogeneous phenotypic shift of VSMCs. A number of miRNAs have been demonstrated to be associated the dysfunction of endothelial cells in aging [13, 23, 40]. However, the mechanism that miRNAs affect phenotypic transition in aging VSMCs is incompletely defined.

In the present study, the expressions of miRNAs in VSMCs from old rats (oVSMCs) and young rats (yVSMCs) were compared using miRNA microarray, and 28 downregulated and 34 upregulated miRNAs were identified in oVSMCs compared with those of the yVSMCs. We focused on miR-542-3p on the basis of microarray detection and demonstrated that miR-542-3p inhibited osteogenic transition of yVSMCs induced by β-glycerophosphate (β-GP) through targeting bone morphogenetic protein 7 (BMP7). Our study suggests that, in aging rats, the decrease in miR-542-3p expression promotes the osteogenic transition in VSMCs by targeting BMP7.

## Materials and methods

### Cell culture

Thoracic aortas of rats were harvested and VSMCs were isolated as previously described [31]. Cells from passage 3–5 were used for the experiments.

### Microarray analysis

Approximate 1.5 × 10^5^ cells were plated into 12-well plates precoated with collagen I (Thermo Fisher Scientific). After synchronization for 24 h by serum starvation, VSMCs were cultivated in DMEM supplied with 10% FBS. After 2 days, total RNA was extracted as previously described [28]. Samples were labeled, hybridized, and scanned (Kang-Chen Bio-tech, Shanghai, China), and images were imported into GenePix Pro 6.0 software for grid alignment and data extraction.

### Small RNA transfection

Approximately 2.5 × 10^5^ VSMCs were plated into 6-well plates. After 24 h, transfection was performed with 50-nM anti-miR-542-3p (yVSMCs), 50-nM pre-miR-542-3p (oVSMCs), or the associated negative controls, using Lipofectamine RNAiMAX (Invitrogen, Carlsbad, CA, USA). After 12 h, fresh DMEM was added and RNA extraction was performed 24 h later.

### Quantitative reverse transcription real-time PCR

To quantify the miR-543-3p expression, 1.0 μg of total RNA was reverse transcribed using a TaqMan miRNA Reverse Transcription kit (Applied Biosystems, Shanghai, China). miR-542-3p expression was measured using a miR-542-3p TaqMan microRNA assay (Applied Biosystems). The expression of miR-543-3p was quantified using the 2^−ΔΔCt^ relative quantification method. The relative expression levels of miR-542-3p were determined by normalizing to that of housekeeping gene U6.

To detect the expression of smooth muscle–related genes and calcification-related genes, quantitative real-time PCR (qRT-PCR) was conducted following the reverse transcription. The primer sequences used for SM22α, calponin, OPN, Runx2, and glyceraldehyde-3-phosphate dehydrogenase (GAPDH) are listed in Table 1. The relative mRNA expressions were determined by normalizing to that of the housekeeping gene GAPDH.

**Table 1:** Primer sequences for real-time PCR

Genes	5′-3′	Primers	Product Size (bp)	Reference sequence
*SM 22α*	Forward Reverse	CCCGCCCTCCATGGTCTTCAAG GCCAAACTGCCCAAAGCCATTAC	165	NM_031549.1
*Calponin*	Forward Reverse	CGGGCACCAAGCGGCAGATCT CCGGGGTCAGGCAGTACTTGGGA	165	NM_031747.1
*OPN*	Forward Reverse	CATCAGAGCCACGAGTTTCA TCAGGGCCCAAAACACTATC	274	NM_012881.2
*Runx2*	Forward Reverse	CAGACCAGCAGCACTCCATA CAGCGTCAACACCATCATTC	178	NM_001278483.1
*GAPDH*	Forward Reverse	AAGTTCAACGGCACAGTCAAGG CGCCAGTAGACTCCACGACATA	139	NM_017008.4

### Luciferase reporter construction and luciferase activity assay

BMP7 mRNA was predicted to be a target of miR-542-3p by the use of TargetScan and Miranda. The 3′-UTR of BMP7 containing the predicted targeting site for miR-542-3p was synthesized (BMP7 WT). A mutated sequence was synthesized as well by changing 2 nucleotides (BMP7 MUT). The 3′-UTR of the luciferase reporter containing the BMP7 WT sequence or the BMP7 MUT sequence was inserted into the pmirGLO vector (Promega, Beijing, China). The pre-miR-542-3p was synthesized and inserted in the pSilencer4.1 vector (pSilencer4.1-miR-542-3p, Invitrogen).

For the luciferase assay, about 10^5^ HEK293 cells were seeded into 12-well plates. Cells were then co-transfected with BMP7 WT vector or BMP7 MUT vector, and pSilencer4.1-miR-542-3p or pSilencer4.1 empty vector. Luciferase activity assay was performed after 24 h, using the Dual-Luciferase Reporter System (Promega).

### Lentivirus construction and transduction

Lentivirus was constructed and transduced into yVSMCs to overexpress miR-542-3p or BMP7. Briefly, the coding sequences of target cDNAs were subcloned into the pCDH vector and co-transfected into HEK293T cells with psPAX2 and pMD2.G using Lipofectamine 2000 (Invitrogen). Virus particles were collected from the supernatant 48 h after transfection and used for the infection of yVSMCs. The infected yVSMCs were incubated in growth medium in the presence of puromycin for stable selection. Cells infected by virus particles generated by empty pCDH vector were used as control.

### Calcification induction and Alizarin red staining

To induce calcification, yVSMCs stably expressing miR-542-3p were cultivated in growth medium supplemented with 10-nM β-GP (Sigma-Aldrich; Merck KGaA, Darmstadt, Germany). After a 7-day incubation, yVSMCs were fixed with 95% ethanol for 30 min at room temperature and stained with 1% Alizarin red (Sigma-Aldrich, Merck KGaA) for 15 min. After washing, the calcium deposition was photographed under an inverted phase-contrast microscope (Olympus, Tokyo, Japan). The calcium deposition was quantified by dye elution as previously described [9].

### Western blot

VSMCs lysis was conducted using RIPA lysis buffer (Beyotime, Shanghai, China) supplied with 1% protease inhibitors. The proteins were separated by electrophoresis and transferred onto PVDF membranes by electroblotting for 3 h at 150 mA. Then, the membranes were blocked for 1 h at room temperature in Tris-buffered saline containing 0.1% Tween 20 (TBST) and 5% skim milk. The membranes were incubated with primary antibodies against BMP7 (Abcam, Cambridge, MA), SM22α (Abcam), calponin (Abcam), OPN (Cell Signaling Technology, Danvers, MA, USA), Runx2 (Cell Signaling Technology), and GAPDH (ZSGB-Bio, Beijing, China), and then the HRP-conjugated secondary antibody (MultiSciences, Beijing, China). Chemiluminescent signals were detected and quantified by densitometry using Quantity One Bioanalysis software (Bio-Rad).

### Statistical analysis

Data were presented as means ± standard deviation (SD). For the comparison of groups, Tukey’s multiple comparisons test was used following ANOVA. *P* < 0.05 was considered statistically significant.

## Results

### miRNA expression in oVSMCs and yVSMCs

In our experiment, 260 miRNAs were statistically analyzed using miRNA array. Our results indicated 28 downregulated and 34 upregulated miRNAs in oVSMCs when comparing with yVSMCs (Additional file 1: Table S1 and S2). According to the microarray data, miR-542-3p expression was reduced 14-fold in oVSMCs. Given that the effect of miR-542-3p in VSMC senescence and its relationship with cardiovascular diseases has barely known, we focused on miR-542-3p in this study.

**Fig. 1:**
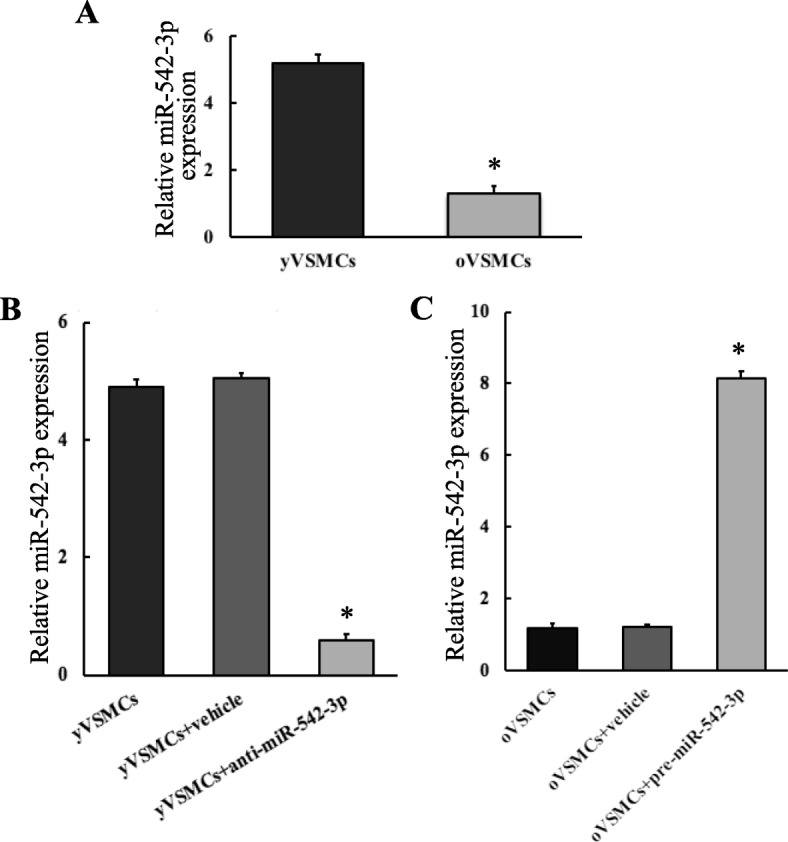
miR-542-3p expression in yVMSCs and oVMSCs detected by qRT-PCR. **a** miR-542-3p expression was approximately downregulated 4-fold in oVSMCs comparing with yVSMCs. *n* = 3. Versus yVSMCs, *p < 0.05. **b** Transfection of anti-miR-542-3p into yVSMCs (yVSMCs+anti-miR-542-3p) significantly downregulated the miR-542-3p expression. *n* = 3. Versus yVSMCs and yVSMCs transfected with its associated negative control (yVSMCs+vehicle) *p < 0.05. **c** Transfection of pre-miR-542-3p into oVSMCs (oVSMCs+pre-miR-542-3p) significantly upregulated the miR-542-3p expression. *n* = 3. Versus oVSMCs and oVSMCs transfected with its associated negative control (oVSMC+vehicle) *p < 0.05

To confirm the miRNA microarray data, qRT-PCR was used to explore the miR-542-3p expression levels in yVSMCs and oVSMCs. We found that miR-542-3p was four times lower expressed in oVSMCs versus that in yVSMCs (Fig. 1a). Furthermore, anti-miR-542-3p transfection into yVSMCs resulted in a significant downregulation of miR-542-3p expression (Fig. 1b), while transfection of pre-miR-542-3p into oVSMCs gave rise to a significant upregulation of miR-542-3p (Fig. 1c). These data suggested that miR-542-3p was abundant in yVSMCs whose expression was decreased within the senescence of VSMCs.

### miR-542-3p inhibited osteogenic transition in yVSMCs induced by β-GP

**Fig. 2:**
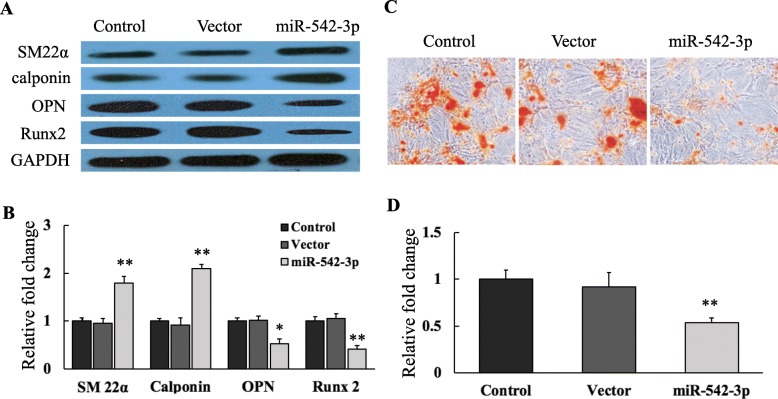
miR-542-3p inhibited osteogenic transition in yVSMCs induced by β-GP. Control yVSMCs, yVSMCs infected with an empty virus vector, and yVSMCs stably expressing miR-542-3p (miR-542-3p) were induced to calcify by β-GP. **a** After 7 days of induction, expressions smooth muscle markers (SM22α and calponin) and osteogenic markers (OPN and Runx2) were investigated by Western blot. GAPDH was used as loading control. **b** Quantification of protein expressions by densitometric analysis. *n* = 3. Versus control and vector yVSMCs, *p < 0.05 and **p < 0.01. **c** After β-GP induction for 7 days, calcium deposition was detected by Alizarin red staining. **d** Quantification of calcium deposition. *n* = 3. Versus control and vector yVSMCs, **p < 0.01

To explore the effect of miR-542-3p on the osteogenic transition, miR-542-3p was overexpressed in yVSMCs using lentivirus, and the miR-542-3p-expressing cells were cultured in growth medium containing 10 nM β-GP for 7 days to induce calcification. Western blot data showed that after β-GP induction, the expressions of the smooth muscle markers SM22α and calponin were significantly higher in yVSMCs overexpressing miR-542-3p than those of yVSMCs (control) and yVSMCs infected with empty virus (vector) (Fig. 2a and b). After β-GP induction, the osteogenic markers OPN and Runx2 were expressed in control and vector yVSMCs. However, overexpressing miR-542-3p in yVSMCs decreased the OPN and Runx2 expressions induced by β-GP (Fig. 2a and b).

Calcium deposition assay by Alizarin red staining was performed to morphologically confirm osteogenic differentiation. After β-GP induction, obvious calcium nodules were observed in control and vector yVSMCs, while in miR-542-3p–overexpressing yVSMCs, the calcium nodules were much fewer than in the other two groups (Fig. 2c). We further quantified the calcium deposition by eluting the Alizarin red and determining the optical density of the eluates. Consistent with the morphological data (Fig. 2c), these results demonstrated that the calcium deposition in miR-542-3p yVSMCs was suppressed when compared with that in control and vector yVSMCs (Fig. 2d). In general, these findings demonstrated that miR-542-3p overexpression could restrain the osteogenic phenotype of yVSMCs induced by β-GP. Thus, our results suggested that miR-542-3p might play a crucial role in regulating the osteogenic transition of VSMCs with senescence.

### miR-542-3p targeted BMP7

**Fig. 3:**
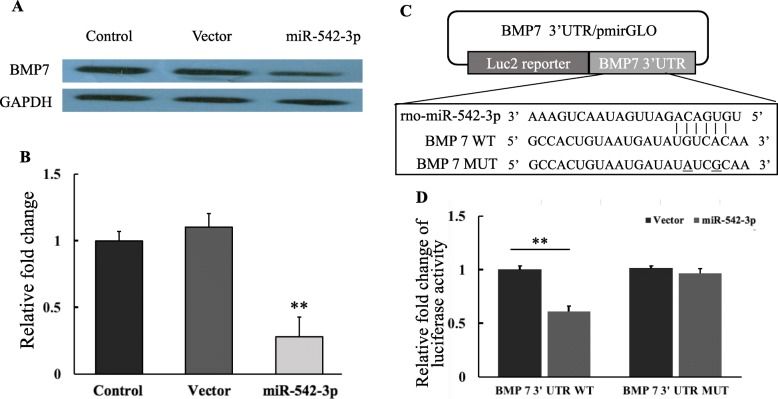
miR-542-3p targeted BMP7. **a** After 7 days of induction by β-GP, the protein expression of BMP7 was detected by Western blot. yVSMCs overexpressing miR-542-3p (miR-542-3p) expressed an obviously lower level of BMP7 than control yVSMCs and yVSMCs infected with an empty virus vector. **b** Quantification of BMP7 expression by densitometric analysis. The relative expression of BMP7 was normalized to that of GADPH. *n* = 3. Versus control and vector group, **p < 0.01. **c** BMP7 3′-UTR constructs used in luciferase assay. **d** Effect of miR-542-3p overexpression on a dual luciferase reporter plasmid containing the BMP7 3′-UTR was analyzed. HEK293T cells were co-transfected with either the BMP7 WT or BMP7 MUT or an empty vector and miR-542-3p (miR-542-3p). Renilla luciferase activity was quantified and normalized to firefly luciferase activity. *n* = 3. Versus vector group, **p < 0.01

BMPs were discovered by their capability to induce bone formation. To explore the potential targets of miR-542-3p involved in osteogenic transition of VSMCs, Targetscan and Miranda databases were used, and BMP7 was identified as a potential target. Therefore, BMP7 expression and its correlation with miR-542-3p were examined in yVSMCs induced by β-GP. As shown in Fig. 3a and b, after 7 days of induction by β-GP, yVSMCs overexpressing miR-542-3p expressed a significantly lower level of BMP7 than control yVSMCs and yVSMCs infected with empty virus, suggesting that miR-542-3p could suppress BMP7 expression in yVSMCs induced by β-GP, and that BMP7 is a potential target of miR-542-3p.

To confirm whether BMP7 is targeted by miR-542-3p, a luciferase reporter assay was performed using BMP7 3′-UTR constructs (Fig. 3c). The 3′-UTR of the luciferase reporter was generated containing BMP7 WT sequence or BMP7 MUT sequence. The luciferase assay showed that the overexpression of miR-542-3p strongly inhibited the luciferase activity in the BMP7 WT group but did not the BMP7 MUT group (Fig. 3d), which demonstrated that BMP7 is a direct target of miR-542-3p.

### miR-542-3p regulated osteogenic transition by targeting BMP7

**Fig. 4:**
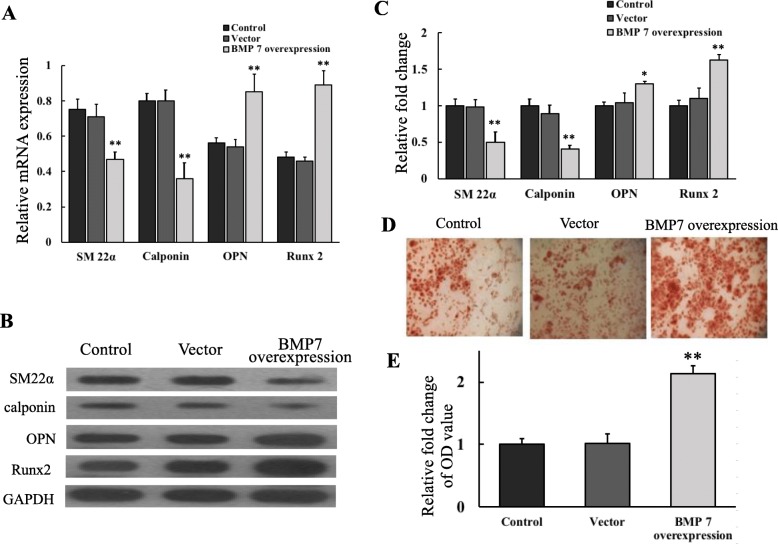
miR-542-3p regulated osteogenic transition by targeting BMP7. yVSMCs overexpressing miR-542-3p (miR-yVSMCs) were transduced with BMP7-overexpressing lentivirus or control empty virus. After 7 days of induction by β-GP, the mRNA (**a**) and protein (**b**) expression levels of smooth muscle markers (SM22α and calponin) and osteogenic markers (OPN and Runx2) were detected. **c** Quantification of protein expression levels by densitometric analysis. **d** Calcium deposition detected by Alizarin red staining. **e** Quantification of calcium deposition. Control: miR-yVSMCs; Vector: miR-yVSMCs transduced with control empty virus; BMP7 overexpression: miR-yVSMCs transduced with BMP7-overexpressing lentivirus. *n* = 3. Versus control and vector groups, *p < 0.05, **p < 0.01

To explore whether the targeting of miR-542-3p to BMP7 regulating the osteogenic transition of VSMCs, yVSMCs stably overexpressing miR-542-3p (miR-yVSMCs) were used for transduction with BMP7-overexpressing lentivirus or control empty virus. The expression of smooth muscle markers SM22α and calponin was significantly downregulated by overexpressing BMP7 in miR-yVSMCs, while the mRNA expression of osteogenic markers OPN and Runx2 was strongly increased in BMP7-overexpressing miR-yVSMCs when compared with that of the control miR-yVSMCs or miR-yVSMCs transduced with control vector (Fig. 4a). Consistent with the mRNA expression data, the Western blot results also demonstrated that overexpressing BMP7 in miR-yVSMCs decreased the protein expression levels of the smooth muscle markers SM22α and calponin, and increased the protein expression levels of the osteogenic markers OPN and Runx2 (Fig. 4b and c).

Alizarin red staining was performed to examine calcium deposition. Calcium deposition was significantly higher in miR-yVSMCs overexpressing BMP7 compared with that of the control miR-yVSMCs or miR-yVSMCs transduced with control vector (Fig. 4d and e). Together, these results demonstrated that overexpressing BMP7 in miR-yVSMCs reversed miR-542-3p’s suppression of the osteogenic transition of yVSMCs, suggesting that miR-542-3p regulated osteogenic transition of VSMCs via targeting BMP7.

## Discussion

In this study, by miRNA microarray, we identified 28 downregulated and 34 upregulated miRNAs in oVSMCs compared with yVSMCs. Among these miRNAs, miR-542-3p, whose expression level was 14 folds downregulated in oVSMCs, was selected for further study. Our results showed that miR-542-3p suppressed the osteogenic differentiation of yVSMCs induced by β-GP. BMP7 was found to be directly targeted by miR-542-3p, and overexpressing BMP7 in miR-542-3p–expressing yVSMCs could reverse miR-542-3p’s inhibition of osteogenic differentiation. Our findings indicated that downregulation of miR-542-3p promotes the osteogenic transition of VSMCs in the aging rat via targeting BMP7.

Cardiovascular disease is a major cause of death worldwide and the biological mechanisms of its development remain unclear. In recent years, the roles of miRNAs in regulating cell functions have been proved in the cardiovascular system, such as cardiomyocytes, endothelial cells, fibroblasts, and smooth muscle cells [33]. miRNAs are non-coding RNAs that inhibit protein translation and/or anneal to mRNAs and promote their cleavage. Accumulating reports have proved the involvement of miRNAs in pathological changes of the cardiovascular system [1, 12, 33].

Cardiovascular diseases are a prevailing issue in aging patients. Vascular senescence has a close connection with a broad spectrum of cardiovascular diseases, with the characteristics of endothelial dysfunction and phenotypic transition of smooth muscle cells, resulting in increased vascular stiffness and increased thickness of vascular walls. It has been reported that the age-associated phenotypic transition of VSMCs is a crucial contributor to vascular remodeling [17, 25]. However, the mechanism that drives phenotypic transition of VSMCs with aging remains unclarified. In this study, using RNAs extracted from the in vitro cultured VSMCs, we explored the miRNA expression profile by microarray. Owing that rat aortas are composed of many kinds of cells, including VSMCs, endothelial cells, and fibroblasts, to avoid the contamination of RNA from endothelial cells and fibroblasts, we extracted the RNAs extracted from the in vitro cultured VSMCs instead from the aortas, which may be a drawback of our study. By microarray, we investigated miRNA expressions in yVSMCs and oVSMCs and identified 28 miRNAs downregulated in oVSMCs, including miR-196a-5p (~ 107-fold), miR-196b-5p (~ 48-fold), miR-542-3p (~ 14-fold), and miR-363-5p (~ 8-fold). miR-196a-5p and miR-196b-5p were reported to be involved in regulating cancer progression [26, 27, 36, 37]. Thus, miR-542-3p was selected in the present study.

miR-542-3p was previously demonstrated to be involved in osteoblast proliferation and differentiation [14]; however, its function in vascular cells is unclear. In our study, inhibited expression of osteogenic markers and calcium deposition were observed in miR-542-3p-overexpressing yVSMCs, while the expression of smooth muscle markers was upregulated, indicating a pivotal role of miR-542-3p in regulating osteogenic transition of VSMCs. Certain miRNAs have been reported to induce switching of VSMCs to the synthetic phenotype, including miR-let-7 g [32], miR-132 [5], miR-145 [19], miR-32 [20, 21], and miR-22 [36, 37]; some of which, for example miR-145 and miR-32, were identified to regulate osteogenic switching of VSMCs. However, miRNAs involved in the age-associated phenotypic conversion of VSMCs have not been completely identified. miR-542-3p has been proved to suppress cancer cell growth [20, 21, 30]. Our previous work showed that the downregulation of miR-542-3p promoted neointimal formation in aging rats [28]. In the present study, we showed that miR-542-3p regulates the osteogenic transition of VSMCs, and with aging, downregulated miR-542-3p was associated with the calcification of the vascular wall.

BMPs are a group of proteins originally discovered to have the capability in inducing osteogenesis and chondrogenesis. There are more than 20 members in BMP family, and some family members, especially BMP2 and BMP7, have been shown to induce osteoblast differentiation and bone formation *in vivo* and *ex vivo* [35]. BMP signaling was also found to be associated with vascular diseases [4, 8] and vascular calcification [11, 38]. In our study, BMP7 was confirmed to be a target of miR-542-3p. Consistent with our study, it was reported that miR-542-3p suppresses osteoblast cell proliferation and differentiation by targeting BMP7 signaling [14]. The miRNAs miR-22 [22] and miR-22-3p [34] were also demonstrated to target BMP7. Additionally, using an online database miRDB (http://mirdb.org), we predicted the potential targets of rno-miR-542-3p, and identified 328 predicted targets for miR-542-3p (Additional file 1: Table S3), which could further help us to understand the role of miR-542-3p in VSMCs.

Generally, in this study, overexpression of miR-542-3p inhibited BMP7 expression in yVSMCs and osteogenic differentiation of yVSMCs, which can be reversed by overexpressing BMP7 in miR-542-3p–overexpressing yVSMCs, suggesting that miR-542-3p regulates osteogenic transition of VSMCs via targeting BMP7. BMP7 is extensively expressed in various tissues and is connected to the development and pathological changes of bone, renal, and ocular systems, as well as the central nervous system [15]. Although BMP7 has been demonstrated to play important role in osteogenesis [35], its role in the osteogenic transition of VSMCs and vascular calcification has not been well documented. Instead, some studies related BMP2 to vascular calcification by its role in regulating osteoblast differentiation of VSMCs [6, 18, 39]. Thus, our study demonstrated novel signaling of BMP7, regulating the osteogenic transition of VSMCs and vascular calcification.

## Conclusions

**Fig. 5:**
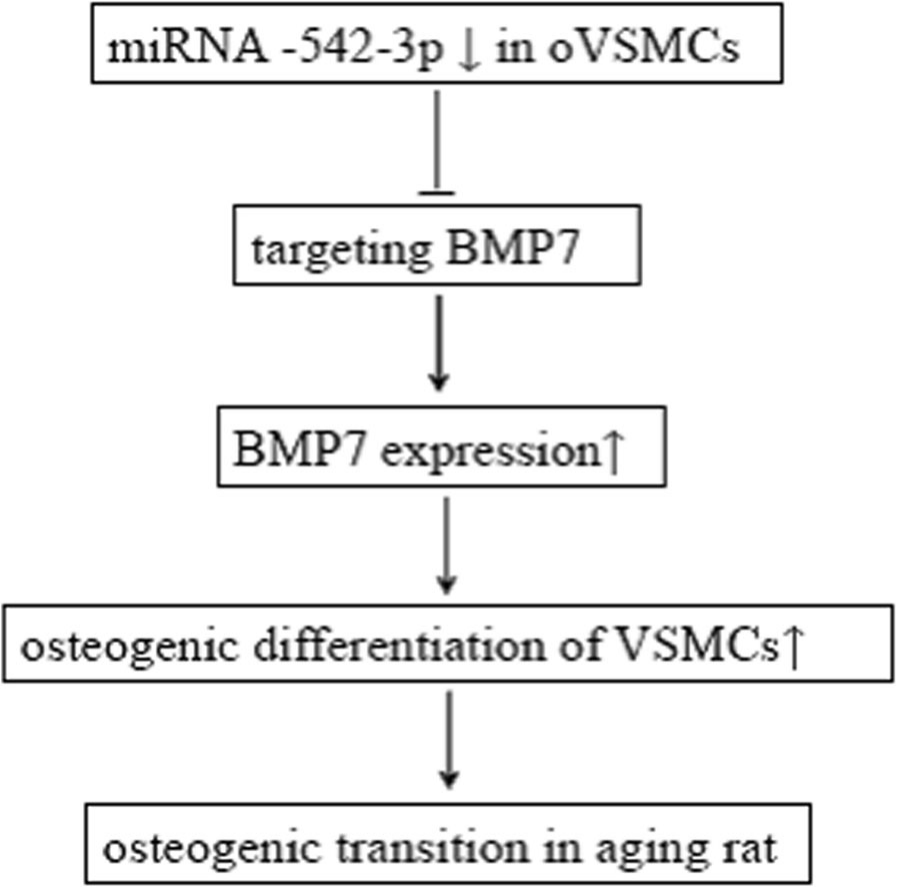
A proposed schematic diagram of miR-542-3p regulating the osteogenic transition of VSMCs in aging rats. MiR-542-3p expression is downregulated in oVSMCs. As a target of MiR-542-3p, the expression of BMP7 is upregulated which results in the osteogenic differentiation of VSMCs, suggesting the role of miR-542-3p in the vascular osteogenic transition in aging rat

We demonstrated that miR-542-3p was differentially expressed in oVSMCs and yVSMCs and demonstrated that miR-542-3p overexpression prohibited BMP7 expression and osteogenic differentiation in yVSMCs induced by β-GP, which can be reversed by overexpressing BMP7 in miR-542-3p–overexpressing yVSMCs. A proposed schematic diagram of miR-542-3p regulating the osteogenic transition of VSMCs in aging rats is shown in Fig. 5. Together, our results suggested that miR-542-3p regulates the osteogenic transition of VSMCs in aging rats by targeting BMP7. These findings are helpful for a better understanding of the role of miRNAs in regulating the osteogenic transition of VSMCs in aging.

## Supplementary information

**Additional file 1: Table S1.** oVSMCs vs yVSMCs 2.0 fold upregulated miRNAs. **Table S2.** oVSMCs vs yVSMCs 2.0 fold downregulated miRNAs. **Table S3.** Predicted targets of miR-542-3p.

## Data Availability

Not applicable.
